# Controlling Endemic *Neospora caninum*-Related Abortions in a Dairy Herd From Argentina

**DOI:** 10.3389/fvets.2019.00446

**Published:** 2019-12-12

**Authors:** Horacio Lagomarsino, Agustín Scioli, Alejandro Rodríguez, Joaquín Armendano, Franco Fiorani, Ángel Bence, Joaquín García, Yanina Hecker, Ignacio Gual, Germán Cantón, Anselmo Odeón, Carlos Campero, Dadín Moore

**Affiliations:** ^1^Private Veterinary Practitioner, Venado Tuerto, Argentina; ^2^Animal Production Department, National Institute of Agrarian de Technology, Balcarce, Argentina; ^3^Faculty of Agricultural Sciences, National University of Mar del Plata, Balcarce, Argentina; ^4^National Research Council, Buenos Aires, Argentina; ^5^Faculty of Veterinary Sciences, National University of the Center of the Province of Buenos Aires, Tandil, Argentina

**Keywords:** bovine, control, management, protozoa, reproductive

## Abstract

After diagnosis of endemic abortions due to neosporosis in a commercial dairy farm, routes of *Neospora caninum*-transmission were evaluated in order to choose the best strategy for reducing its seroprevalence and related abortions. Fifty two dam-calf pairs were bled at parturition. Additionally, 22 female calves were also sampled at regular 3 month intervals until 18–22 months. *N. caninum* specific antibodies were assayed by IFAT. Serum samples were tested at a dilution 1:25 for calves before colostrum intake and heifers before mating and 1:100 for multiparous cows. Only serum samples from IFAT seropositive cattle involved in the evaluation of the routes of transmission were assessed by a commercial IgG avidity ELISA. Seropositive cows or heifers were artificially inseminated with semen from Hereford bulls. The progenies from these female animals were sent to a feed lot to produce meat. Different generalized linear models (GLM) were used to study the relationship between abortion, age category, and serostatus. Seropositive heifers were more likely to have a record of abortion (OR 2.7; 95% CI 1.6–4.7). Vertical transmission frequency was 55.5% (5 seropositive calves/9 seropositive cows). Horizontal transmission was 22.7% (5 female calves seroconverted at least one time/22 females calves sampled during 24 months) and these 5 female calves had low avidity. In heifers, both seroprevalence and abortion rates decreased from 22.1 and 8.4% of 475 in 2009 to 6.1 and 4.3% of 578 in 2015, respectively (*p* < 0.01). Over 5 years, *N. caninum*-seroprevalence and the related abortions in heifers decreased after the control strategy was assessed.

## Introduction

The coccidian parasite named *Neospora caninum* causes a costly abortigenic disease in cattle worldwide (1). The parasite persists in cattle mainly by vertical transmission (2, 3) but horizontal transmission involving the definitive hosts has been also documented (3–5). Usually, both routes of transmission are associated with two epidemiological patterns of abortions. While endemic abortions are associated with frequent vertical transmission, epidemic abortions have been documented when horizontal transmission is involved (4). Although both beef and dairy cattle can suffer *N. caninum* related abortions, livestock production system and genetic risk factors may increase the manifestation of the clinical disease in dairy cattle rather than in beef cattle (6, 7).

Unfortunately, no treatment or vaccines are yet available to control bovine neosporosis (8). Indeed, the only way to control the disease is by interrupting the parasite cycle (8, 9). At farm biosecurity level, many strategies have been suggested: (1) testing replacement and purchased cattle; (2) prevention of transmission from definitive hosts; (3) rodent control; and (4) prevention of any immunosuppressive factors such as virus infection, ingestion of mycotoxins or any cause of stress which could lead to reactivation of chronic infection and vertical transmission. Also, some reproductive management practices have been proposed to control transmission. Embryo transfer is an effective way to prevent vertical transmission from seropositive dams (10, 11). Interestingly, artificial insemination (AI) using semen from beef bulls (specifically Limousin and Belgian Blue) reduced the rate of *N. caninum* related abortions of seropositive dairy dams (12, 13). Endocrine patterns associated to placental well-being, particularly high levels of pregnancy-associated glycoprotein (PAG)-2, decrease the risk of abortion due *N. caninum* in dairy cows (14). Furthermore, the lowest PAG-2 concentrations have been recorded in *N. caninum* seropositive pure-breed Holstein Friesian pregnancies (7).

Before any control strategy is adopted, the identification of the most frequent routes of transmission and presence of definitive hosts or wildlife reservoirs must be evaluated (8, 9). Moreover, in farms where high prevalence is recorded, the only profitable control strategy is do not breed heifers born from seropositive cows (15). Recently, a successful control of bovine neosporosis by using beef-breed semen in seropositive dams has been described (16). Although seropositive cattle were not culled, the control management included retesting seronegative animals every year and the breed of the beef semen was not reported (16). Since studies of controlling the disease in commercial dairy herds with diagnosis of endemic neosporosis are scarce, here we provide the information collected from a commercial dairy farm where a similar strategy was applied.

## Materials and Methods

### Herd Management

The animals belonged to a dairy farm located at Córdoba province, Argentina (33°52′ 51.96″S, 62°50′ 30.84″W). There were approximately 3,000 dairy cattle housed on 2,000 ha of grasslands. The herd was composed by different dairy crossbreeds (Jersey, Holstein, and Swiss Fleckvieh). Reproductive management was based on two calving seasons: early spring and autumn.

Calves and heifers were raised under semi-extensive grazing conditions. Heifers were grazed on pasture (stocking rate = 2 heads/ha). Heifers were first mated at 18 months of age.

One month before first mating, heifers underwent a routine clinical and genital examination. Immunization using 2 doses of a commercial vaccine (Biogénesis-Bagó™, Argentina) against *Leptospira* spp., bovine herpes virus type 1 (BoHV-1) and bovine viral diarrhea virus (BVDV), was performed.

Daily heat was detected during breeding season, and artificial insemination (AI) was performed, following natural service for 1 month with bulls. Pregnancy was tested by ultrasonography. All animals were tuberculosis and brucellosis free. There were three *N. caninum* seropositive dogs in the property.

### Diagnosis of Reproductive Problems

During 2009, 13.1% out of 746 heifers aborted; differential serological diagnosis was performed in dams that aborted, using BVDV and BoHV-1 seroneutralization, *Leptospira* spp. microscopic agglutination test (MAT), and *N. caninum* indirect fluorescent antibody test (IFAT) (11). Additionally, all heifers and cows were bled the same year in order to calculate the association of abortion and *Neospora* serostatus (17).

### Control Strategy (Rationale and Sampling)

Frequencies of routes of transmission were estimated in the herd in order to establish the best strategy to reduce the *N. caninum*-seroprevalence and related abortions. Therefore, precolostrum blood samples were collected immediately after calving from 52 calves (30 males and 22 females) and their dams (within 24 h postpartum) by jugular venipuncture. Additionally, 22 female calves were also sampled at regular 3 month-intervals until 24 of age. Serum samples were obtained after centrifugation and then stored at −20°C until analysis.

Once the management to control bovine neosporosis began, a simple blood sample was obtained from heifers before their mating in order to perform the serological test. Only seropositive animals having one of the following “events”: (1) abortion; (2) mastitis; (3) digital dermatitis; (4) low milk production; (5) low body condition score were culled (16). All data regarding reproduction, health events, nutrition, and milk production was managed using the software PROTAMBO MASTER (DIRSA SH, Gonnet, Buenos Aires, Argentina). *N. caninum*-seropositive cows and heifers (without any other “event”) were AI with semen from Hereford bulls. The crossbred progenies from these dams were raised in a feedlot for meat production and therefore female crossbred calves were not selected as future dams.

### Serology

#### Parasites and Antigen Slide Preparation

Parasite growing and antigen slide preparation were performed as previously mentioned (18).

#### Indirect Fluorescent Antibody Test (IFAT)

Specific antibodies were assayed by IFAT (19). Precolostrum serum and heifers before mating serum samples were tested at a dilution 1:25 (20). Meanwhile, multiparous cow serum samples were tested at a dilution of 1:100 (18).

#### Avidity Enzyme-Linked Immunosorbent Assay (ELISA)

Only serum samples from IFAT seropositive cattle were assessed by a commercial IgG avidity ELISA (CIVTEST, Hipra BOVIS Neospora^TM^, Spain) in order to evaluate routes of transmission (21). Serum samples were assessed ss recommended by the manufacturers. Briefly, samples were analyzed in duplicate, and the mean value of the optical density (OD) was converted into a relative index per cent (RIPC) as follow: (OD sample–OD negative control)/(OD positive control–OD negative control) × 100. For those samples with an RIPC higher than 6, the antibody avidity index was calculated: (OD sample diluted 1/25–OD sample diluted 1/100)/(OD sample diluted 1/25 and incubated with urea–OD sample diluted 1/100 incubated with urea). The interpretation was: high avidity ≤ 1; intermediate avidity = between 1 and 2; ≥2 low avidity.

### Statistical Analysis

Generalized linear models (GLM) were used, assuming a binomial distribution of the explanatory variable and a logit link function. As first step, the relationship between abortion (response variable), bovine category (heifers or cows), *N. caninum* seropositivity and their interactions (explanatory variables) were analyzed.

Secondly, the frequency of vertical and horizontal transmissions was characterized. Vertical transmission was calculated as follow: number of calves having specific antibodies before colostrum intake/seropositive dams × 100. Frequency of horizontal transmission was calculated as percentage of seronegative precolostrum female calves that had seroconversion until 18–22 months of age.

The effect of the application of the control strategy was verified by three GLMs: (1) to determine the reduction of *N. caninum* seropositivity (variable response) throughout time (explanatory variable), (2) to determine the reduction of the probability of abortion (variable response) over time, according to the bovine category (heifers or cows) and its possible interactions (explanatory variables); and (3) to determine the reduction of the probability of abortion (variable response) by using Hereford semen in different categories over time and its possible interactions (explanatory variables). Model significance was assessed by using likelihood ratio tests. Odds ratio and 95% profile likehood (LR) confidence intervals (CI) were estimated from the resulting logistic model. Analysis were performed in R version 3.4.2 (22) and a 0.05 was used as significance level (https://www.R-project.org/). When necessary, Hosmer-Lemeshow test was used to determine the goodness of fit of the models.

## Results

### Diagnosis of Reproductive Problems

During 2009, serological approaches showed that *N. caninum* was the cause of endemic abortions in the dairy farm. The abortion event was explained due to a significant interaction between serostatus and category being *N. caninum*-seropositive heifers more likely to have a record of abortion (OR: 2.7; CI 95% 1.6–4.7) than seronegative heifers (Table 1). Indeed, the probability of abortion in *N. caninum*-seropositive heifers was 28.3% (CI 95% 22.7–34.9) (*p* < 0.01). Similarly, there was a significant association between abortion and serostatus in cows (*p* < 0.01) (Table 1). On the other hand, differential diagnosis performed in 17 aborted dams did not provide evidence of other abortigenic pathogens (BVDV, BoHV-1, or *Leptospira* spp) responsible of reproductive losses at the herd level.

**Table 1:** Serological diagnosis of *N. caninum* related abortions in heifers and cows from a dairy herd.

**Category**	**Abortion % (*****n*****)**	
	**Seropositive**	**Seronegative**
Cow	14.1 (389)	8.1 (2.026)
Heifer	28.3 (208)	7.2 (538)
Total	19.1 (597)	7.9 (2.564)

### Route of *Neospora* Transmission Frequencies

Vertical transmission frequency was 55.5% (5 seropositive calves were born from 9 seropositive cows). Five out of these 9 seropositive cows had high avidity antibodies and transmitted the disease congenitally to 3 calves; in contrast, the other 4 cows having low avidity antibodies delivered 2 seropositive calves. Horizontal transmission was confirmed in 5 out of the 22 female calves (22.7%) as they seroconverted at least at one sampling time during 24 months. All these 5 female calves had low avidity antibodies suggesting postnatal exposure to the protozoa.

### Seroprevalence and Abortion Rate in Heifers After Applying the Control Strategy

**Figure 1:**
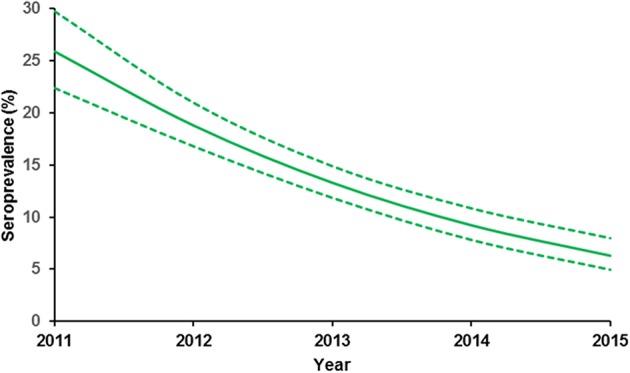
*N. caninum* seroprevalence probability values estimated in dairy heifers over time after applying the control management in 2011. The estimated values and CI_95%_ were obtained from GLM.

**Figure 2:**
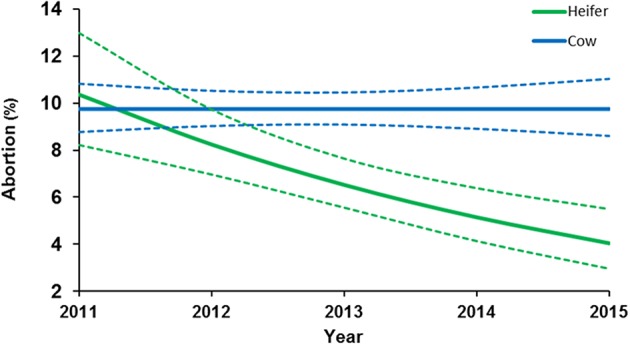
Abortion probability values on dairy heifers and cows over time after applying the control management in 2011. The estimated values and CI_95%_ were obtained from GLM.

During 2011–2015, *N. caninum*-seroprevalence and abortion rate significantly decreased from 28 to 6.1% and 13.3 to 4.3%, respectively (Table 2). In that period, “seropositivity” was associated with abortion (OR: 5.9; CI 95% 4.1–8.5) (*p* < 0.01) but the “year” was associated with protection from abortion (OR: 0.66; CI 95% 0.6–0.7) (*p* < 0.01). The estimated seroprevalence after applying the control strategy is shown in Figure 1. Abortion rate remains stable in multiparous cows but there was a significant reduction in the rate of abortions in heifers (OR: 0.83; CI 95% 0.8–0.9) (*p* < 0.01) (Figure 2).

**Table 2:** Descriptive data about prevalence and *N. caninum*-related abortion in dairy heifers after by selective culling and artificial insemination (AI) using Hereford semen on seropositive dairy cattle.

	**Year**				
	**2011**	**2012**	**2013**	**2014**	**2015**
Prevalence (%)	28.0	19.3	15.9	8.9	6.1
Abortion (%)	13.3	4.9	8.6	3.7	4.3
Total (*n*)	375	409	523	327	578

Surprisingly, the probability of abortion was higher by using Hereford semen in different categories evaluated over time (Table 3). Seropositive cattle either heifers or cows, independent of a given year, were more likely to suffer an abortion than those animals with the same serostatus but inseminated with dairy semen (OR: 1.5; CI 95% 1.0–2.2) (*p* < 0.03).

**Table 3:** Results from logistic regression to estimate effect of using Hereford semen on seropositive dairy cattle.

**Response variable**	**Explanatory variable**	*P*-value^*^	OR^*^	LR 95% CI^*^
Abortion	Year	0.69		
	Category	0.40		
	Hereford semen	0.03	1.51	1.0–2.2

* *Odds ratio (OR) estimates and profile likelihood (LR) 95% confidence intervals for being serologically positive to N. caninum to abortion over time*.

## Discussion

Once bovine neosporosis is confirmed as the main cause of abortions, different alternatives are available for reducing the negative impact in the production system (9). Our approach was based in an initial differential diagnosis of the cause of the elevated abortion rate on this dairy farm. Clear evidence of endemic *N. caninum* related abortions was confirmed affecting mainly heifers. Abortion risk in herds with endemic neosporosis has been well-documented to be higher in heifers than in multiparous cows (23). Indeed, here we recorded a decreasing *N. caninum*-seroprevalence and related abortions in heifers rather than multiparous cows. Also, decreasing the seroprevalence was associated with reduction of endemic abortions even when seropositive cattle were not culled (16). Retesting seronegative animals every year was necessary for that successful control management but the cost-benefit balance was not reported. On the other hand, “do not breeding replacements from seropositive cows” was the only financially attractive strategy in farms with a high *N. caninum* prevalence (15). After a proper diagnosis, testing only heifers every year could be a profitable option. Definitively, an economical evaluation at farm level must be performed before any option is recommended.

Testing calves before colostrum intake is unpractical but not impossible in extensive dairy production systems having two calving seasons. This approach was essential in order to check routes of transmission and therefore to evaluate whether any strategy may fail. As expected transplacental transmission was over 50% (2) but horizontal transmission was also recorded, as previously reported (3, 4, 21). Because the recorded high postnatal exposure, farm biosecurity measures were also recommended. Indeed, feeding dogs with commercial food and avoiding contact with cows were measures also implemented. However, the impact of such measures could not be evaluated. On the other hand, the consequences of postnatal exposure before mating are still unknown.

Although there are several seroepidemiological studies showing that beef cattle are less susceptible to both *Neospora* infection and abortion than dairy cattle (6, 12, 24), our results suggest an increased frequency of abortion when using Hereford semen in seropositive heifers or cows of the breeds Jersey, Holstein, and Swiss Fleckvieh crossbreeds. These findings must be taken with caution since placenta functions in crossbreed pregnancies can be enhanced by using beef bull semen (12). PAG levels, which have been associated with a healthy materno fetal interfase, were found to be higher in crossbreed pregnancies (7). It is probable that the heterosis was already established by using Jersey, Holstein, and Swiss Fleckvieh crossbreeds before 2009 in the herd, so, insemination with beef semen did not show any effect. Similarly to that reported by Sala et al. (16) the exclusion of the progeny obtained from seropositive cattle from the dairy system was a successful control strategy to reduce both seroprevalence and *N. caninum*-related abortions. Moreover, the meat productive performance of Hereford crossbreed was higher than that in dairy pure crossbreeds having a positive economic return (data no shown).

Even in absence of treatment or vaccine for prevention, here we provide additional evidence of controlling *N. caninum*-related abortions in a commercial dairy herd with endemic bovine neosporosis. Recording data and evaluating many scenarios, including production of bovine meat, were important in order to control bovine neosporosis in a commercial dairy herd under semi-extensive grazing conditions.

## Data Availability Statement

The datasets generated for this study are available on request to the corresponding author.

## Ethics Statement

The animal study was reviewed and approved by Animal Ethics Committee at National Institute of Agricultural Technology, Argentina. Written informed consent was obtained from the owners for the participation of their animals in this study.

## Author Contributions

HL, DM, CC, and AO conceived and supervised the study. HL and DM designed this study. ÁB, JG, IG, and AR performed field work. FF and YH conducted laboratory testing. JA and AS analyzed data. DM and GC wrote the manuscript. HL, CC, and AO contributed to the study design and provided funding acquisition, access to animals, and laboratory oversight. All authors read and approved the manuscript.

### Conflict of Interest

The authors declare that the research was conducted in the absence of any commercial or financial relationships that could be construed as a potential conflict of interest.

## References

[1] Reichel MP Ayanegui-Alcérreca MA Gondim LFP Ellis JT. What is the global economic impact of *Neospora caninum* in cattle – The billion dollar question. Int J Parasitol. (2013) 43:133–42. 10.1016/j.ijpara.2012.10.022 23246675

[2] Anderson ML Reynolds JP Rowe JD Sverlow KW Packham AE Barr BC. Evidence of vertical transmission of *Neospora* sp infection in dairy cattle. J Am Vet Med Assoc. (1997) 210:1169–72. 9108925

[3] Moré G Bacigalupe D Basso W Rambeaud M Beltrame F Ramirez B. Frequency of horizontal and vertical transmission for *Sarcocystis cruzi* and *Neospora caninum* in dairy cattle. Vet Parasitol. (2009) 160:51–4. 10.1016/j.vetpar.2008.10.081 19070964

[4] McAllister MM Björkman C Anderson-Sprecher R Rogers DG. Evidence of point-source exposure to *Neospora caninum* and protective immunity in a herd of beef cows. J Am Vet Med Assoc. (2000) 217:881–7. 10.2460/javma.2000.217.881 10997162

[5] Dijkstra T Barkema HW Eysker M Wouda W Evidence of postnatal transmission of *Neospora caninum* in Dutch dairy herds. Int J Parasitol. (2001) 31:209–15. 10.1016/S0020-7519(00)00160-0 11239942

[6] Moore DP Campero CM Odeón AC Posso MA Cano D Leunda MR. Seroepidemiology of beef and dairy herds and fetal study of *Neospora caninum* in Argentina. Vet Parasitol. (2002) 107:303–16. 10.1016/S0304-4017(02)00129-2 12163242

[7] García-Ispierto I Serrano-Pérez B Almería S Martínez-Bello D Tchimbou AF de Sousa NM. Effects of crossbreeding on endocrine patterns determined in pregnant beef/dairy cows naturally infected with *Neospora caninum*. Theriogenology. (2015) 83:491–6. 10.1016/j.theriogenology.2014.10.013 25459029

[8] Dubey JP Schares G Ortega-Mora LM. Epidemiology and control of neosporosis and *Neospora caninum*. Clin Microbiol Rev. (2007) 20:323–67. 10.1128/CMR.00031-06 17428888 PMC1865591

[9] McAllister MM. Diagnosis and control of Bovine Neosporosis. Vet Clin North Am Food Anim Pract. (2016) 32:443–63. 10.1016/j.cvfa.2016.01.012 27161392

[10] Baillargeon P Fecteau G Paré J Lamothe P Sauvé R. Evaluation of the embryo transfer procedure proposed by the International Embryo Transfer Society as a method of controlling vertical transmission of *Neospora caninum* in cattle. J Am Vet Med Assoc. (2001) 218:1803–6. 10.2460/javma.2001.218.1803 11394835

[11] Campero CM Moore DP Odeón AC Cipolla AL Odriozola E. Aetiology of bovine abortion in Argentina. Vet Res Commun. (2003) 27:359–69. 10.1023/A:1024754003432 14509450

[12] López-Gatius F Santolaria P Yániz JL Garbayo JM Almería S. The use of beef bull semen reduced the risk of abortion in *Neospora* seropositive dairy cows. J Vet Med B. (2005) 52:88–92. 10.1111/j.1439-0450.2004.00818.x 15752268

[13] Almería S López-Gatius F García-Ispierto I Nogareda C Bech-Sàbat G Serrano B. Effects of crossbreed pregnancies on the abortion risk of *Neospora caninum*-infected dairy cows. Vet Parasitol. (2009) 163:323–9. 10.1016/j.vetpar.2009.04.026 19464119

[14] García-Ispierto I Almería S Serrano B de Sousa NM Beckers JF López-Gatius F. Plasma concentrations of pregnancy-associated glycoproteins measured using anti-bovine PAG-2 antibodies on day 120 of gestation predict abortion in dairy cows naturally infected with *Neospora caninum*. Reprod Domest Anim. (2013) 48:613–8. 10.1111/rda.12134 23228018

[15] Häsler B Stärk K Gottstein B Reist M. Epidemiological and financial considerations for the control of *Neospora caninum* on Swiss dairy farms. Schweiz Arch Tierheilkd. (2008) 150:273–80. 10.1024/0036-7281.150.6.273 18605018

[16] Sala G Gazzonis A Boccardo A Coppoletta E Galasso C Manfredi MT. Using beef-breed semen in seropositive dams for the control of bovine neosporosis. Prev Vet Med. (2018) 161:127–33. 10.1016/j.prevetmed.2018.10.024 30466653

[17] Thurmond MC Hietala SK Strategies to control *Neospora* infection in cattle. Bovine Pract. (1995) 29:60–3.

[18] Moore DP Konrad JL San Martino S Reichel MP Cano DB Méndez S. *Neospora caninum* serostatus is affected by age and species variables in cohabiting water buffaloes and beef cattle. Vet Parasitol. (2014) 203:259–63. 10.1016/j.vetpar.2014.04.011 24792747

[19] Dubey JP Hattel AL Lindsay DS Topper MJ. Neonatal *Neospora caninum* infection in dogs: isolation of the causative agent and experimental transmission. J Am Vet Med Assoc. (1988) 193:1259–63. 3144521

[20] Venturini MC Venturini L Bacigalupe D Machuca M Echaide I Basso W. *Neospora caninum* infections in bovine foetuses and dairy cows with abortions in Argentina. Int J Parasitol. (1999) 29:1705–8. 10.1016/S0020-7519(99)00143-5 10608457

[21] Rodríguez AM Maresca S Cano DB Armendano JI Combessies G Lopéz-Valiente S. Frequency of *Neospora caninum* infections in beef cow-calf operations under extensive management. Vet Parasitol. (2016) 219:40–3. 10.1016/j.vetpar.2016.02.002 26921037

[22] R Core Team. R: A Language and Environment for Statistical Computing. R Foundation for Statistical Computing, Vienna, Austria (2017). Available online at: https://www.R-project.org/.

[23] Thurmond MC Hietala SK. Effect of congenitally acquired *Neospora caninum* infection on risk of abortion and subsequent abortions in dairy cattle. Am J Vet Res. (1997) 58:1381–5. 9401685

[24] Santolaria P Almería S Martínez-Bello D Nogareda C Mezo M Gonzalez-Warleta M. Different humoral mechanisms against *Neospora caninum* infection in purebreed and crossbreed beef/dairy cattle pregnancies. Vet Parasitol. (2011) 178:70–6. 10.1016/j.vetpar.2010.12.018 21216103

